# Dichoptic Metacontrast Masking Functions to Infer Transmission Delay in Optic Neuritis

**DOI:** 10.1371/journal.pone.0163375

**Published:** 2016-10-06

**Authors:** Maximilian Bruchmann, Catharina Korsukewitz, Julia Krämer, Heinz Wiendl, Sven G. Meuth

**Affiliations:** 1 Institute of Medical Psychology and Systems Neuroscience; University of Münster, Münster, Germany; 2 Institute for Biomagnetism and Biosignal Analysis; University of Münster, Münster, Germany; 3 Department of Neurology; University of Münster, Münster, Germany; Monash University, AUSTRALIA

## Abstract

Optic neuritis (ON) has detrimental effects on the transmission of neuronal signals generated at the earliest stages of visual information processing. The amount, as well as the speed of transmitted visual signals is impaired. Measurements of visual evoked potentials (VEP) are often implemented in clinical routine. However, the specificity of VEPs is limited because multiple cortical areas are involved in the generation of P1 potentials, including feedback signals from higher cortical areas. Here, we show that dichoptic metacontrast masking can be used to estimate the temporal delay caused by ON. A group of 15 patients with unilateral ON, nine of which had sufficient visual acuity and volunteered to participate, and a group of healthy control subjects (N = 8) were presented with flashes of gray disks to one eye and flashes of gray annuli to the corresponding retinal location of the other eye. By asking subjects to report the subjective visibility of the target (i.e. the disk) while varying the stimulus onset asynchrony (SOA) between disk and annulus, we obtained typical U-shaped masking functions. From these functions we inferred the critical SOA_max_ at which the mask (i.e. the annulus) optimally suppressed the visibility of the target. ON-associated transmission delay was estimated by comparing the SOA_max_ between conditions in which the disk had been presented to the affected and the mask to the other eye, and vice versa. SOA_max_ differed on average by 28 ms, suggesting a reduction in transmission speed in the affected eye. Compared to previously reported methods assessing perceptual consequences of altered neuronal transmission speed the presented method is more accurate as it is not limited by the observers’ ability to judge subtle variations in perceived synchrony.

## Introduction

Optic neuritis (ON) is the most frequent cause of subacute visual loss in young adults. Patients suffer from a loss of vision developing in hours to days that is associated with painful eye movement. Often fogging of vision and changes in color perception are also described. Classical ON is associated with multiple sclerosis or seen as a clinically isolated syndrome at the early stage. Aside from its association with multiple sclerosis (MS), ON occurs in other autoimmune inflammatory diseases, especially in neuromyelitis optica spectrum diseases (NMOSD) or systemic inflammatory diseases with CNS involvement. In addition to clinical exams, MRI can show gadolinium enhancement of the optic nerve; laboratory analyses are used to exclude differential diagnosis and confirm the autoimmune origin of inflammation [1].

For patients, pain and loss of visual acuity are the most prominent symptoms [2]. However, as ON and other neuro-inflammatory diseases not only decrease the amount of transmitted signals but also transmission speed, the question arises, how visual perception is affected if neuronal signals are delayed at the level of the optic nerve. The goal of the present study is to assess the effects of this delay on visual perception.

In clinical practice, visual evoked potentials (VEPs) are used to evaluate the extent of transmission delay in optic neuritis by presenting stimuli either to the affected or unaffected eye and measuring the latency and amplitude of the P1, i.e. the first positive deflection in the evoked signal at occipital sensors around 100–120 ms after stimulus onset. A preserved P1 potential associated with a peak latency delay is seen as characteristic of demyelination. Decrease in amplitude is interpreted as axonal damage [3,4].

VEP latencies can be measured reliably and offer objective estimates of cortical response timing. However, even early VEPs have to be viewed as convoluted results of several neural generators [5,6] and feedback loops [7,8]. Furthermore, while a latency shift of VEPs may reflect altered neural timing (i.e. delayed neural processing) it may also be caused by different response magnitudes of relatively early and late responding cortical sites (i.e. relatively more/less engagement of slower/faster processes). Thus, conclusions drawn from shifted VEP latencies are ambiguous with regard to information about their neural generators.

Another strategy to assess altered neuronal transmission speed is to test patients with temporally sensitive perceptual behavioral tasks: Parsons [9] showed that critical flicker fusion frequency (CFF) was lowered in patients with a history of ON. Similarly, Heron, Regan and Milner [10] measured perceived synchrony of brief flashes of light presented to the affected and unaffected eye. The authors found that the affected eye showed latency increases of up to 110 ms and argued that demyelination alone could not explain such a strong reduction, suggesting that response slowing had to originate at the retinal level. This interpretation is supported by more recent evidence showing that ON causes lesions already at the level of the retina due to retrograde degeneration leading to axonal loss in retinal nerve fiber layers and loss of secondary retinal ganglion cells [11,12].

While the perceived synchrony approach by Heron and colleagues appears very elegant and simple, its precision is limited by the observers’ ability to judge fine-grained deviations from synchrony. In the present study we took a similar approach but instead of obtaining responses based on the temporal comparison of percepts originating from both eyes we employed a metacontrast-masking paradigm. Rather than having subjects judge the temporal delay, this paradigm required judgements of brightness or visibility reduction, which have been shown to be highly sensitive to temporal variations of the stimuli (for an overview see e.g. [13]). Metacontrast masking is a classical experimental paradigm in which the visibility of a briefly presented stimulus (e.g. a uniform gray disk) can be dramatically reduced by a successively presented and spatially surrounding gray annulus. This reduction in visibility can be assessed e.g. by means of subjective ratings on a 5-point scale, and it peaks at a specific stimulus onset asynchrony (SOAs) between disk (henceforth called the target) and annulus (henceforth called the mask). The exact peak SOA strongly depends on experimental and individual factors, but within one experimental setup and within one observer, it yields a robust and precise time stamp of optimal suppression. Metacontrast masking is an especially promising approach for studies of unilateral ON as it can be applied dichoptically, i.e. the target can be presented to the left eye, the mask to the right, and vice versa [13–15]. Under monoptic viewing conditions (i.e. target and mask presented to the same eye) the SOA leading to maximal suppression is typically around 30–80 ms [13,16–19]. Under dichoptic viewing conditions the suppression is typically stronger and peaks at shorter SOAs as compared to monoptic viewing conditions [20], presumably because the effect of binocular rivalry adds to the suppressive effect of the mask at near-simultaneous presentation of the mask [13]. Our goal is to make use of this high temporal sensitivity of metacontrast masking and to employ its dichoptic variant to compare the time stamp of optimal suppression between ON-affected and–unaffected eyes.

A prevailing view on the neuronal mechanisms of metacontrast masking is that the target signal first enters some cortical area X, proceeds to higher levels of processing and then re-enters X at the same moment as the mask signal [7,8,21]. An alternative view states that X integrates signals from a specific system of cortical neuronal representations and a nonspecific reticulo-thalamic system, called perceptual retouch [22–24]. Since the nonspecific process is slower, the mask signal has an advantage of being integrated for perception while the specific target signals have already decayed when nonspecific signals come in. The exact cortical locus of X and the exact mechanisms of the suppressive effects are still under debate, but for the purpose of the present study it is sufficient to assume that there exists some optimal SOA between target and mask at which the mask signal “hits” the target signal on time. With long SOAs the mask misses the target because the mask entered X after target signals have decayed. With short SOAs mask signals may either precede target signals, or may be integrated into one signal, forming the joined percept of target and mask. As neuronal signals need to have a certain temporal extent, the typically obtained U-shaped masking function can be viewed as the result of varying overlap of target and mask signals at X. Its minimum defines the SOA that maximizes this overlap, henceforth called SOA_max_.

Visual masking paradigms have been previously applied to patients with optic neuritis [25] and multiple sclerosis [26,27], the studies either aiming at a general assessment of visual functions [25], or at estimating the degree to which cortical lesions delayed conscious perception of masked stimuli [26,27]. Marx et al. used monocular masking (i.e. all stimuli were presented to one eye). Reuter et al. used binocular masking (i.e. all stimuli were always presented to both eyes). In both monocular and binocular masking, a potential signal processing delay affects target as well as mask signals. Thus, the relative delay between the two signals, and hence the SOA of optimal masking, will not show systematic differences between lesioned and unaffected processing pathways. To gain this information one needs to have the target stimulus processed exclusively in the lesioned pathway and the mask stimulus in the unaffected pathway, or vice versa.

Consequently, we employed dichoptic masking in the present study, that is, we presented the target stimulus to one eye and the masking stimulus to the other. This method yields the optimal temporal delay between target and mask, with only one of the two signals being affected by lesions in the ON-affected optic nerve. The following result pattern should emerge as a consequence of neuronal transmission delay: if the target is presented to the ON-affected and the mask to the unaffected eye, the SOA of optimal masking (SOA_max_) should be increased as compared to the condition in which the target is presented to the unaffected and the mask to the affected eye. In the first case, SOA_max_ is expected to increase, because the delayed target requires an additional head start in order to be “hit on time” by the mask. The reverse rationale applies to the second case: SOA_max_ is expected to decrease because a delayed mask needs to start earlier than an un-delayed mask, in order to hit the target.

## Methods

### Subjects

The control group consisted of eight participants (four female). Their age ranged from 27 to 34 years (M = 29.63, SD = 2.88). Seven subjects were right-handed, one was left-handed. All control subjects had normal or corrected-to-normal vision.

Fifteen patients diagnosed with unilateral optic neuritis were screened for sufficient visual acuity. Monocular near visual acuity was measured using standardized number charts. A line of optotypes was considered to have been read correctly when more than 50% (e.g., 3 of 5) of the optotypes presented have been read correctly. Standard test distance was 35 cm. Visual acuity was given in decimal notations (e.g. 1.0 corresponding to 20/20 Snellen notation). All patients included had a monocular visual acuity of at least 25% (25% corresponding to a Snellen notation of 20/80).

Patients with a history of other medical or psychiatric diseases that may influence perception of visually presented stimuli were excluded from participation. All patients underwent neurologic and ophthalmologic examination including visual acuity measurement and fundoscopy. The group of patients meeting the inclusion criteria consisted of nine participants (four female). Their age ranged from 19 to 56 years (M = 35.27, SD = 10.77). Eight patients were right-handed, one was left-handed. In five patients the left eye was affected by ON, in four the right eye. Visual acuity in the affected eye, as measured by Snellen charts was between 25% and 80% (M = 61.34%, SD = 22.37%). All patients had been treated with methylprednisolone intravenously within one to seven days prior to the experiment. All patients and healthy participants volunteered for participations and gave written informed consent. Only healthy volunteers were rewarded with 9 Euro per hour. All procedures were carried out according to the declaration of Helsinki and were approved by the ethical committee of the medical faculty of the University of Münster.

### Apparatus and stimuli

All stimuli were presented on a Samsung 2233RZ LCD monitor running at 120 Hz and a resolution of 1680 × 1050 pixels. The minimum and maximum luminance of the screen was 0.17 and 131 cd/m^2^, respectively, and the mean luminance was 64.89 cd/m^2^. The experiment was run using MATLAB (Version 2013a, The MathWorks) and the Psychophysics Toolbox (Version 3.0.10; [28–30]).

Participants watched the screen through a mirror stereoscope (Geoscope, Standard Mirror Stereoscope). The stereoscope was mounted to a forehead-and-chin rest with two adjustable matte black tubes to be used as oculars. A matte gray panel positioned vertically between the mirrors, running from the rear opening of the oculars to the front of the monitor assured that light emitted at one side of the display was not visible at the other side. The front opening of the oculars was positioned 60 cm in front of the monitor.

**Fig 1:**
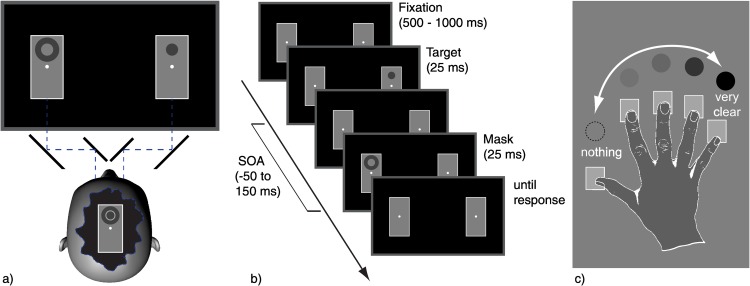
Illustration of stimulus setup and procedure. (a) The display was viewed through a mirror stereoscope. The gray squares were constantly presented to ensure binocular fusion. (b) Example of a trial sequence, here with a target presented to the right eye, and a mask to the left eye, both above the fixation point. Negative SOAs refer to trials where the mask preceded the target. (c) Illustration of the assignment of keys to perceived target contrast. The same figure (with German labels) was part of the on-screen instruction.

As illustrated in Fig 1A, two rectangular medium gray (64.89 cd/m^2^) rectangles surrounded by thin white frames subtending 6° × 12° (width × height) each were presented left and right from the center of the screen, their centers separated by 14°. These frames remained on the screen throughout the experiments to ensure constant binocular fusion.

Stimuli will be described in terms of the Michelson contrast relative to the background, i.e. 99.5% contrast will refer to a black stimulus (0.17 cd/m^2^) and 0% contrast will refer to a stimulus that is as gray as the background.

The target stimulus was a uniform dark gray disk (various contrasts, but always darker than the background, see below) with a radius of 1°. Its center was positioned 2.5° below or above a fixation cross in the center of each frame. The mask was a dark gray annulus, concentric with the disk. Its inner radius was 1.05°, its outer radius was 2.05°. A stimulus (target or mask) was always presented only to one eye, never to both. Fig 1B contains an illustration of the stimuli and a trial sequence.

Responses were recorded with a custom-built external response box with five keys corresponding to each finger of the right hand. A left-hand response box was used for left-handed participants.

### Procedure

Patients and controls underwent slightly different procedures. Basically, the patients were presented with a subset of the experimental conditions used for the controls to reduce measurement time, since we expected the procedure to be more exhausting for patients with optic neuritis. We will therefore describe the procedure for controls first and then highlight the differences to the patients’ procedure.

The participants were seated in front of the stereoscope (see Fig 1A) and were asked to adjust the chin rest, the oculars and the chair to their needs. They were instructed to rate the visibility of dark disks, which could randomly appear either below or above the central fixation mark. They were asked to judge the visibility intuitively and spontaneously by pressing one of the five keys. For illustration purposes instructions contained a drawing of a hand with a dark disk assigned to the pinky finger, a slightly brighter disk assigned to the ring finger, up to a dashed outline of an invisible disk assigned to the thumb (see Fig 1C). They were told that there were no right or wrong answers and were asked to fixate on the centrally presented fixation dot.

To accustom participants with the task and the brief stimulus presentations, we first presented participants with 50 practice trials in which disks were shown with a contrast of 43.5% for 100 ms without being followed by a mask. The targets were presented randomly to the left or right eye, above or below fixation. Next, we presented 50 practice trials (for patients: 160 trials) in which disks were shown with contrasts ranging from 43.5% to 2% for 25 ms, also without being followed by a mask. Again, the targets were presented randomly to the left or right eye, above or below fixation. Before the next 50 practice trials, participants were informed that from now on an annulus would appear additionally to the disk. They were instructed to ignore the annulus and to continue rating the visibility of the target disk. The following 50 practice trials were chosen from the complete set of conditions to be used in the main experiment: targets and masks were always presented at 17% contrast for 25 ms. The possible SOAs were -50, -25, -17, 0, 17, 25, 33, 42, 50, 58, 75, 100, and 150 ms, with negative SOAs referring to forward masking, i.e. the mask preceded the target (Fig 1B). With the same probability as each single SOA (i.e. 6.67%), we presented trials in which either only the target, or only the mask was presented. With equal probability the target could appear either below or above the fixation point (referred to as factor *height*). If both the target and the mask were presented on a trial, they were always presented at the same height. To compare monoptic and dichoptic metacontrast masking we presented the target either to the left or right eye (referred to as *target eye*). Independently of the target eye, the mask could also be presented to the left or right eye (referred to as *mask eye*). With 13 SOAs plus target- and mask-only trials, two heights, two target eyes and two mask eyes we had 120 different experimental conditions. The 50 practice trials were chosen randomly from this set of conditions. In the main experiment, each condition was repeated 14 times. The resulting 1680 trials were presented in randomized order. A break was introduced after every 200 trials. The break lasted at least 30 seconds, after which the participants could initiate the next trial with a key press whenever they felt ready. An experimental session, including instruction and practice trials lasted on average about 90 minutes.

For patients, we checked whether a potential loss of visual acuity significantly lowered the contrast threshold in the affected eye. For this purpose the 160 practice trials, in which the target only was presented at various contrasts to one or the other eye were analyzed. Averaged visibility ratings were compared between affected and unaffected eye for each contrast level. Contrast adjustments would have been made if averaged visibility ratings differed significantly between the affected and unaffected eye at a target contrast of 17%. However, this was not the case in any patient, so both eyes of all patients received the same target and mask contrasts as the controls.

In contrast to controls we did not employ monoptic masking for patients for the sake of reduced measurement duration. Thus targets and masks were always presented to opposite eyes. The possible SOAs were -50, -25, -17, 0, 8, 17, 33, 50, 66, 75, 100, and 150 ms. Again, target- and mask-only trials were presented with a probability equal to each single SOA (i.e. 7.14%, as there was one SOA less for patients than for controls). With 12 SOAs plus target- and mask-only trials, two heights, and two target eyes we had 56 different experimental conditions. With 14 repetitions per condition this resulted in 784 trials. Pauses were introduced after every 120 trials. An experimental session, including instruction and practice trials lasted on average about 60 minutes. In all other aspects, the procedure was identical between patients and controls.

## Results

To analyze the time point of optimal masking (SOA_max_) we followed a procedure used by Bruchmann, Breitmeyer and Pantev [31], i.e. we normalized the subjective ratings first, fitted a masking function based on an inverted log-normal curve and then used the minimum of that function as an estimate of SOA_max_. Specifically, we used the average ratings from target- (*R*_*T*_) and mask-only (*R*_*M*_) trials to normalize the subjective ratings in the following way:

$$R_{n}=\frac{R-R_{M}}{R_{T}-R_{M}}$$

where R refers to the ratings per subject, SOA, height, target eye and mask eye, averaged across 14 measurement repetitions. *R*_*T*_ and *R*_*M*_ refer to the averaged ratings per subject, height, target eye and mask eye. Consequently, *R*_*n*_ = 0 indicates visibility ratings corresponding to an absent target and *R*_*n*_ = 1 indicates visibility ratings corresponding to an unmasked target.

For each subject we averaged the subjective ratings, separately for target and mask eye and for each SOA, but collapsed across targets presented above and below the fixation dot.

After normalization, the data were fit by the following function:

$$R_{n\left(t\right)}=\alpha -\beta ∙e^{-{\left(\frac{\log\left(t\right)-t_{0}}{\delta }\right)}^{2}}$$

As can be seen in Table 1, the data of three patients could not be fitted successfully as indicated by a non-significant amount of explained variance (R^2^) for at least one side of target presentation. These three subjects were excluded from all further analysis.

**Table 1:** Individual Fit results–Main Experiment.

		Target to left eye, mask to right			Target to right eye, mask to left		
ID	affected eye	SOA_max_ (ms)	R^2^	p	SOA_max_ (ms)	R^2^	p
C01	-	29.95	.937	< .001	25.49	.972	< .001
C02	-	21.95	.947	< .001	28.91	.928	< .001
C03	-	29.57	.975	< .001	24.87	.930	< .001
C04	-	29.18	.993	< .001	33.36	.976	< .001
C05	-	45.29	.943	< .001	51.34	.970	< .001
C06	-	33.40	.980	< .001	39.42	.943	< .001
C07	-	18.10	.906	< .001	26.66	.939	< .001
C08	-	33.15	.960	< .001	34.36	.955	< .001
*P01*	*right*	*-22*.*47*	.*176*	.*113*	***67*.*15***	**.*354***	**.*013***
P02	left	**36.17**	**.909**	**< .001**	29.41	.838	< .001
P03	left	**104.66**	**.353**	**.035**	43.02	.448	.009
*P06*	*right*	*24*.*57*	.*127*	.*156*	***-0*.*86***	**.*208***	**.*046***
P07	right	62.15	.700	< .001	**55.96**	**.636**	**.001**
P08	left	**63.51**	**.317**	**.045**	-13.15	.224	.042
P09	right	8.26	.767	< .001	**21.23**	**.781**	**< .001**
P10	right	67.79	.678	.001	**72.13**	**.360**	**.013**
*P11*	*left*	***-9*.*15***	**.*028***	**.*323***	*-38*.*28*	.*000*	.*250*

Individual results of the fit procedure for the eight control subjects (C01 –C08) and the nine patients (P01 –P11). For the patients, the values of the ON-affected eye (i.e. trials in which the target was presented to the affected and the mask was shown to the unaffected eye) are printed in bold. R^2^ denotes the amount of explained variance by the fitted inverted log-normal function. In three patients at least one fit did not lead to a significant amount of explained variance (p > 0.05). These cases, printed in italics, were excluded from all further analyses.

**Fig 2:**
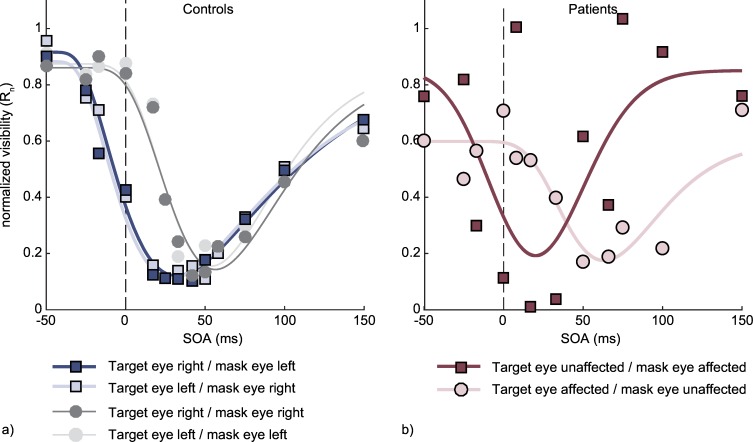
Normalized visibility ratings as a function of SOA, averaged across target presentation side (above or below fixation) and across control subjects (a) or patients (b). The gray lines in panel a) depict the monoptic conditions where targets and masks were presented to the same eye. These conditions were only used in the control group. The colored lines (blue for the control group, red for the patients group) depict the dichoptic conditions, where targets and masks were presented to different eyes. For the control groups we differentiate between the left and red eye whereas for the patients we differentiate between the ON-affected and unaffected eye. Negative SOAs refer to trials in which masks preceded targets, positive SOAs to trials with targets preceding masks. For the purpose of illustration only, the depicted functions were fitted to the visibility ratings after averaging across subjects / patients. All statistical analyses were based on individually fitted functions.

Fig 2 shows the normalized subjective ratings (R_n_) averaged across target positions and subjects, fitted by the function described above. Note, that only for illustrative purposes the function was fitted to the averaged data in the figure. For all statistical analyses the functions were fitted separately for each subject and experimental condition. Individual fit statistics are provided in Table 1.

**Fig 3:**
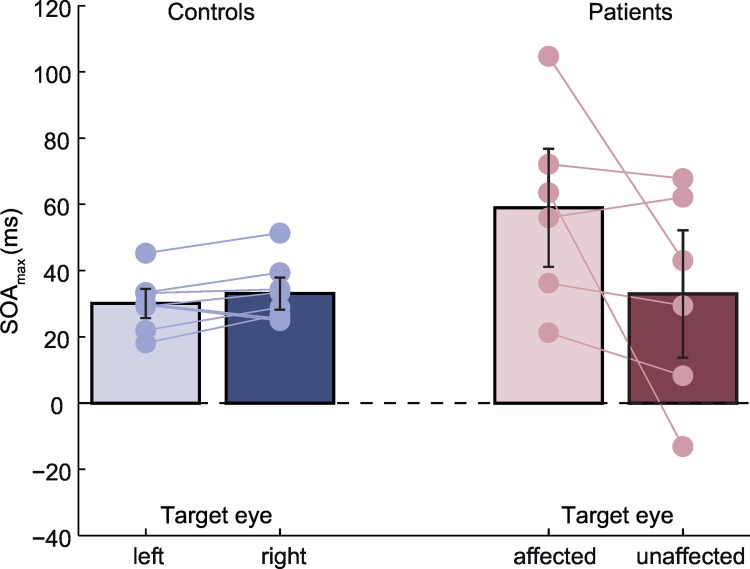
Average SOA_max_ (ms) for the control group (N = 8) and the patients (N = 6), depending on the eye to which the target was presented. The mask was always presented to the opposite eye. Error bars depict the 95% confidence interval of the mean. Each connected pair of circles represents SOA_max_-values from a single subject / patient.

The resulting estimates of SOA_max_ were analyzed by means of a 2 × 2 (group × eye) ANOVA, with repeated measures on the eye-factor. The means of the four conditions are presented in Fig 3.

The ANOVA revealed no significant main effects but a significant interaction, F(1,12) = 5.707, p = 0.034. As Fig 3 indicates, the difference in SOA_max_ between the two eyes was more pronounced in the patient group than in the control group. A planned comparison of the affected and the unaffected eye failed to reach statistical significance, t(5) = 1.859, p = 0.061 (one-sided). Relative to the control group’s average (SOA_max_ = 31 ms), the average SOA_max_ in patients was increased by 28 ms if the target was presented to the affected eye and nearly identical if the target was presented to the unaffected eye.

To judge whether SOA_max_-differences can serve as a diagnostic criterion at the individual level we calculated the 95% confidence interval (CI) of the absolute SOA_max_-differences between left and right eye in the control group (CI = [4.1 ms, 6.5 ms]). Two out of six patients fall within this CI (P07 and P10) and thus would qualify as misses in a diagnostic procedure. The remaining four patients’ SOA_max_-differences fall outside the CI and would be diagnosed correctly. Two out of eight control subjects fall outside the CI and would therefore qualify as false positives. For the remaining six control subjects the diagnosis would be correctly rejected. Given the small sample sizes we regarded Chi-square tests as too unreliable and thus did not analyze the reported contingencies any further.

### Control experiment

**Fig 4:**
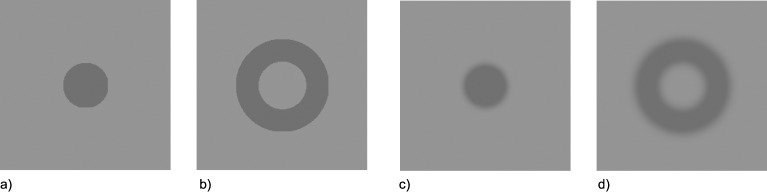
Stimuli used in the control experiment. Stimuli depicted in a) and b) are identical to the stimuli used in the main experiment. Stimuli depicted in c) and d) were created by blurring the contours with a Gaussian kernel while keeping overall stimulus energy (i.e. the average brightness of the display) constant.

A possible caveat for interpretation of the results is the patients’ residual loss of visual acuity, which could have caused a blurred representation of the stimulus contours. It could be argued that the observed results are at least in part caused by spatially distorted stimulus signals, rather than temporally delayed signals. More specifically, if ON-patients perceived comparably blurred edges, their subjective visibility judgments may have been based on the stimulus surface rather than its contours. It has been shown that judging contour properties yields smaller SOA_max_ estimates than judging overall surface brightness [32,33]. Since we did not instruct the participants to specifically judge contour or surface properties we cannot tell whether patients chose different response criteria than control subjects. To test this alternative interpretation we conducted a control study where we mimicked potential vision loss in four healthy participants (two female, mean age = 28 years, three naïve observers plus author MB) by dichoptically presenting one randomly chosen eye with blurred targets and masks (see Fig 4) and the other eye with un-blurred stimuli. Targets and masks were never presented to the same eye on a given trial.

Apart from choosing a slightly adapted set of SOAs for the four control subjects, the apparatus and procedure was identical to the patients’ procedure. This also included the same instructions, which did not emphasize any specific strategy with respect to responding to either the contours or the surfaces of targets.

**Fig 5:**
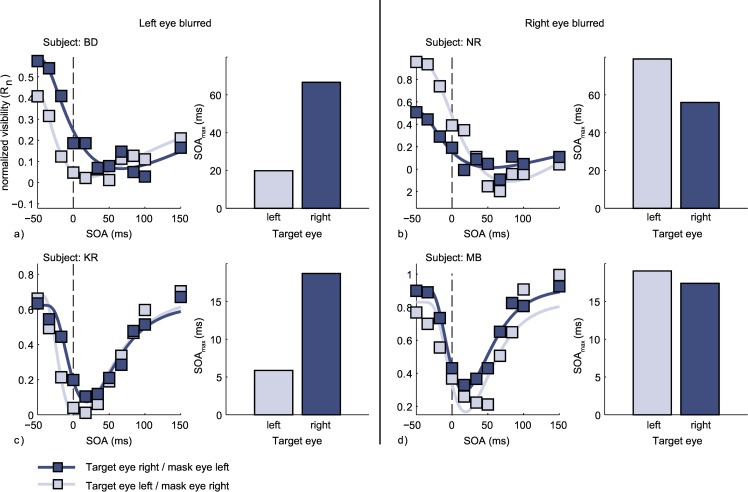
Normalized visibility ratings observed in the control experiment as a function of SOA for four subjects. The control experiment featured only dichoptic conditions, in which targets and masks were presented to different eyes. The two subjects depicted in the left half of the figure, the left eye received blurred stimuli to mimic loss of visual acuity. The two subjects depicted on the right, the right eye received blurred stimuli. The bar charts show the temporal position of the individual minima (SOAmax).

As can be in Fig 5, in all four cases SOA_max_ was lower when a blurred target was masked by an un-blurred annulus than when an un-blurred target was masked by a blurred annulus. Individual statistics are shown in Table 2. The control experiment therefore showed that the pattern of results observed in ON-patients is unlikely to be caused by blurred contours.

**Table 2:** Individual Fit results–Control Experiment.

		Target to left eye, mask to right			Target to right eye, mask to left		
ID	blurred eye	SOA_max_ (ms)	R^2^	p	SOA_max_ (ms)	R^2^	p
BD	L	**19.83**	**.968**	**< .001**	66.63	.956	< .001
KR	L	**5.87**	**.939**	**< .001**	18.70	.951	< .001
NR	R	78.98	.972	< .001	**55.97**	**.911**	**< .001**
MB	R	19.03	.795	< .001	**17.38**	**.969**	**< .001**

Individual results of the fit procedure for the four subjects participating in the control experiment. The values of the eye receiving blurred targets are printed in bold. R^2^ denotes the amount of explained variance by the fitted inverted log-normal function.

## Discussion

Optic neuritis significantly altered the time course of metacontrast masking. By presenting targets and masks dichoptically, we obtained masking functions for conditions in which either the target signal or the mask signal was transmitted through the affected optic nerve. We showed that the SOA that lead to maximal target suppression was greater when the target signal travelled through the affected and the mask through the unaffected nerve compared to the opposite stimulation paradigm. We interpret this as a slowdown of signal transmission within the affected optic nerve. This interpretation is based on a rationale shared by several masking theories, namely that the mask signal, which is triggered later than the target signal (SOA > 0 ms), “hits” the target signal at some point of processing because the target signal has either returned to a previous processing stage [7,8] or contains signal components that persist longer than the stimulus, e.g. a slowly processed sustained signal [34] or a neuronal after-discharge [35]. In all cases an optimal SOA can be found that leads to a maximal impact of the mask signal onto the target signal. All these theories further state that the locus of signal interruption is cortical. The important consequences for the interpretation of the present data are that a transmission delay introduced prior to cortical signal processing should increase the optimal SOA if only the target signal was delayed, and decrease the optimal SOA if only the mask signal was delayed. Our results showed an increase due to delayed target processing by about 28 ms. We did not observe a symmetrical decrease due to delayed mask processing, which could result from an inaccurate SOA_max_ estimation due to the small sample size, but might as well indicate that the mask signal was delayed to a lesser degree than the target signal. The latter interpretation rests on the tentative assumption that the more peripheral retinal locations occupied by the mask may be on average less affected by ON than the more central areas corresponding to the target location.

The control experiment further indicated that loss of visual acuity in the affected eye might have exerted an additional influence on the masking function, however in a direction opposing the proposed transmission delay: if the lesion caused a blurred representation of the stimulus contours, SOA_max_ can be expected to decrease for targets presented to the affected eye. In our view, this finding can be explained by the stimuli’s spatial frequency spectrum. In a previous study [31] we contrasted targets with various spatial frequencies and found prolonged estimates of SOA_max_ whenever a low-frequency mask was used in combination with a high-frequency target. This result reflects the relatively stronger engagement of fast-conducting, low-frequency tuned transient channels triggered by blurred stimuli compared to sharp-edged stimuli. Thus, we conclude that our presented results cannot be explained by decreased visual acuity in patients. In fact, the control experiment implies that if patients processed blurred contours in the affected eye, we underestimated the actual processing delay induced by optic neuritis.

Since there are different methods to assess ON-associated signal delays, we will now review and compare them to the present method. We will focus on the specificity, reliability and clinical practicability of these techniques.

### Visual evoked potentials

Halliday and colleagues were the first to report delayed VEPs in patients with ON [3]. The average shift of the P1 peak latency was 35 ms in the affected compared to the unaffected eye. It has further been shown that while P1 amplitude attenuations only persist during the acute phase of the disease, P1 latency prolongation is present up to two years after disease onset [36]. The main advantage of VEP measurements is certainly their objectivity. Furthermore, once clinical routines in recording VEPs are established, P1 latencies can be measured very efficiently. However, although the P1 component is usually the earliest large deflection in the EEG signal it should not be viewed as the first purely feedforward wave of cortical processing. Cortical source modeling approaches have shown that the P1 reflects a convolution of many cortical sources [5,6] and is already modulated by feedback from higher areas [7]. Consequently, the specificity of P1 latency shifts remains uncertain. Nevertheless, VEP recordings remain a very useful clinical tool to measure prolonged processing latencies efficiently and objectively and in some reports they were used to monitor treatment responses [37].

### Perceptual tests

Measurements of perceptual consequences of delayed visual processing also have a long tradition. They involve estimates of the critical flicker fusions frequency (CFF; [9]), judgements of perceived synchrony [10] and visual masking paradigms (25–27).

Measurements of CFF have been shown to exhibit abnormalities in patients with multiple sclerosis (MS) with good reliability [38]. Surprisingly, Daley et al. report that abnormal CFFs were observed independently of whether the visual system was affected by demyelination or not. It could be argued that unnoticed, subclinical affections of the visual system were responsible for abnormal CFFs. In this case, however, one would expect at least a reduced CFF abnormality in MS patients without clinical affection of the visual system. Daley et al. show that, while the degree of CFF abnormality is correlated with the neurological grade of MS, it is identical in patients with and without visual pathway demyelination. This appears to be a limiting factor for the application of CFF measurements in patients with ON, as altered CFFs may not be indicative of transmission changes in the optic nerve.

Judgment of perceived synchrony of two stimuli presented with various delays to both eyes separately [10] appears to be the most straightforward approach to detect whether one eye shows slower signal transmission than the other. Heron and colleagues observed an average delay of 44 ms in the affected eye, thus a roughly three times longer delay than the one we observed. Additionally, control subjects showed an average difference of 14 ms, with the left eye being slower in about 75% of the measurements. In our data, the control subjects showed an average difference of 5.3 ms (95% CI = [4.1 ms, 6.5 ms]) in SOA_max_ between the left and right eye. Again the average difference in healthy controls is about three times larger in the study of Heron et al. compared to our study. It is difficult to tell from the present data which estimate is the more accurate one. However, as the authors themselves discuss, their estimated difference appears implausibly large given what is known about conduction speeds of neuronal signals in the optic nerve. They conclude that the observed delays of up 110 ms might also be caused at the retinal level [10], which has been shown to be affected by ON through retrograde degeneration [11,12]. Similar to the study of Heron and colleagues, our dichoptic masking method also cannot differentiate between delays caused at the level of the retina and at the level of the optic nerve. We argue, however, that the precision of synchrony judgments rests on the assumption that neuronal delay, introduced at the level of the retina and / or optic nerve, is linearly related to the perceived delay at the level of conscious decision making. Human time perception depends on many factors, such as the intensity or contrast of the stimulus (e.g. [39,40]) and is subject to many illusions (for an overview see [41]). It might therefore be disadvantageous to rely on subjective synchrony judgments in order to estimate neuronal delays. We propose that our masking approach overcomes this problem by asking subjects to report perceived stimulus contrast. On its own, perceived contrast is neither objective, nor indicative of neuronal delays. In fact, it is probably much more indicative of the spatial visual deficits caused by ON. However, the variable of primary interest in the masking approach is not perceived contrast but SOA_max,_ reflecting the dependence of perceived contrast on subtle temporal variations (i.e. target-to-mask SOA). We assume that response criteria do not change systematically with SOA, as the SOA is chosen randomly on each trial from a set of SOAs that is barely discriminable (i.e. at least close to SOA_max_ participants are unlikely to know whether the SOA was 20, 40, or 60 ms). Under the assumption that response criteria are constant over the range of SOAs, SOA_max_ can be viewed as an objective measure although it relies on subjective ratings.

### Considerations for a clinical application of the dichoptic metacontrast method

Our study should be regarded as a proof of principle, showing that subtle processing delays are reflected in the temporal properties of the metacontrast masking curve. Compared to VEPs used in clinical routine, our method offers a more precise localization of transmission delay although being time consuming. The whole measurement took about one hour per patient. Patients were treated with methylprednisolone intravenously for ON, however one would expect rather an amelioration of visual function, thus a decrease of transmission delay. Effects of steroids on masking function were not examined in this study and could be the aim of future projects. Although the group statistics in a relatively small sample show statistically significant differences, the reliability of an individual diagnosis could not be judged due to the small sample size. Descriptively, two false positive (out of eight control subjects) and two false negative (out of six patients) diagnostic decisions indicate that the current paradigm is probably not sufficiently reliable on the individual level. A reasonable approach to optimize the procedure would be to find the SOA_max_ via adaptive measurements, or by the method of adjustment, asking patients to set the SOA manually to a level where target visibility is lowest. We claim that optimizations of the presented method could prove to be a cost efficient way to add valuable diagnostic information about altered transmission speeds caused by optic neuritis.
